# Application of the ITS2 Region for Barcoding Medicinal Plants of Selaginellaceae in Pteridophyta

**DOI:** 10.1371/journal.pone.0067818

**Published:** 2013-06-27

**Authors:** Wei Gu, Jingyuan Song, Yuan Cao, Qingwen Sun, Hui Yao, Qinan Wu, Jianguo Chao, Juanjuan Zhou, Wenda Xue, Jinao Duan

**Affiliations:** 1 College of Pharmacy, Nanjing University of Chinese Medicine, Nanjing, People’s Republic of China; 2 Institute of Medicinal Plant Development, Chinese Academy of Medical Sciences, Peking Union Medical College, Beijing, People’s Republic of China; 3 Department of Pharmacy, Guiyang College of Traditional Chinese Medicine, Guiyang, People’s Republic of China; University of Milano Bicocca, Italy

## Abstract

**Background:**

Selaginellaceae is a family of nonseed plants with special evolutionary significance. Plants of the family Selaginellaceae are similarly shaped and easily confused, complicating identification via traditional methods. This study explored, for the first time, the use of the DNA barcode ITS2 to identify medicinal plants of the Selaginellaceae family.

**Methodology/Principal Findings:**

In our study, 103 samples were collected from the main distribution areas in China; these samples represented 34 species and contained almost all of the medicinal plants of Selaginellaceae. The ITS2 region of the genome was amplified from these samples and sequenced using universal primers and reaction conditions. The success rates of the PCR amplification and sequencing were 100%. There was significant divergence between the interspecific and intraspecific genetic distances of the ITS2 regions, while the presence of a barcoding gap was obvious. Using the BLAST1 and nearest distance methods, our results proved that the ITS2 regions could successfully identify the species of all Selaginellaceae samples examined. In addition, the secondary structures of ITS2 in the helical regions displayed clear differences in stem loop number, size, position, and screw angle among the medicinal plants of Selaginellaceae. Furthermore, cluster analysis using the ITS2 barcode supported the relationship between the species of Selaginellaceae established by traditional morphological methods.

**Conclusion:**

The ITS2 barcode can effectively identify medicinal plants of Selaginellaceae. The results provide a scientific basis for the precise identification of plants of the family Selaginellaceae and the reasonable development of these resources. This study may broaden the application of DNA barcoding in the medicinal plant field and benefit phylogenetic investigations.

## Introduction

**Figure 1:**
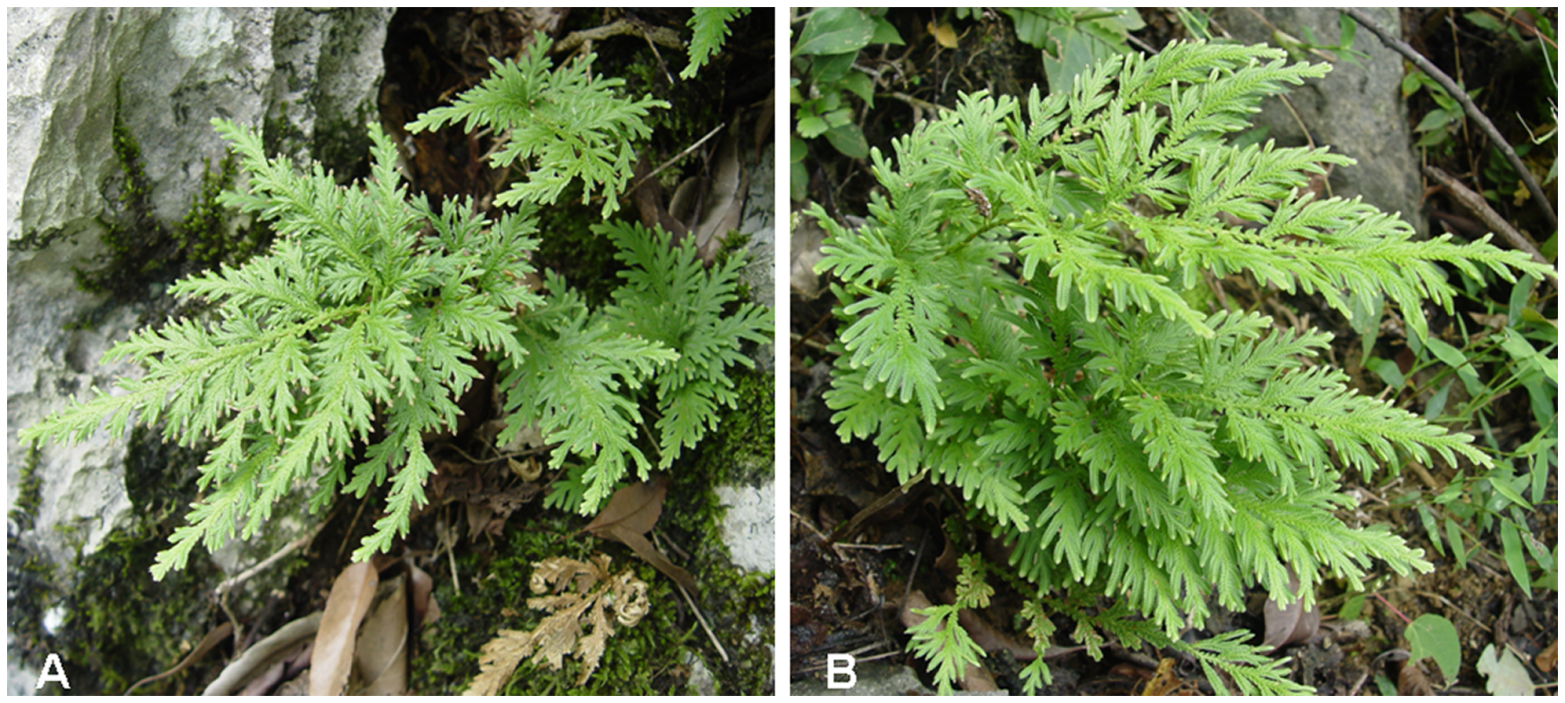
Plant morphology of medicinal plants of the family Selaginellaceae in their natural habitats. A-*S. involvens*, B-*S. moellendorffii*.

The Selaginellaceae family belongs to class Lycopodiopsida in the Pteridophyta division; this family has only one genus, *Selaginella* (spikemoss), which diverged from Euphyllophyta [1]. There are approximately 700 species in the Selaginellaceae family, which are distributed throughout the world, with approximately 50 species located in China; of these species, more than 20 species have medicinal value [2]. Selaginellaceae is an important family with special evolutionary significance. Selaginellaceae differs from other families within Lycopodiopsida in post-transcriptional gene regulation, including small RNA regulation of repetitive elements, the absence of the tasiRNA pathway and extensive RNA editing of organellar genes [1]. Phylogenetic analysis has shown that the *TPS*-like genes of *Selaginella moellendorffii* (*SmMTPSLs*) are more closely related to microbial *TPSs* than other plant *TPSs*. The presence of two distinct types of *TPSs* in the *S. moellendorffii* genome raises the possibility that the *TPS* genes in other plant species may have more than one evolutionary origin [3]. In Selaginellaceae, some species have evolved desiccation tolerance (DT); *S. lepidophylla* appears poised to tolerate desiccation in a constitutive manner by using a wide range of metabolites with some inducible components [4]. Furthermore, the medicinal plants of Selaginellaceae not only have the detoxification, hemostatic, and blood circulation-promoting activities traditionally associated with medicinal plants [5], but also have antitumor [6], antibacterial [7], hypoglycemic [8], and immunoregulatory functions [9]. Therefore, these plants are of increasing interest to domestic and foreign pharmaceutical companies as well as the botany field in general. The main active components of the medicinal plants of Selaginellaceae include biflavones [10], selaginellins [7], phenylpropanoids [11], alkaloids [12], and others. Plants of the Selaginellaceae family usually differ in chemical components and have different activities. These differences could affect the safety and clinical efficacy of the resulting medicines. Thus, differentiating medicinal plant species in the Selaginellaceae family is critical to ensure quality and therapeutic efficacy. However, plants of the family Selaginellaceae are small in size, similar in shape (Figure 1), and easily confused [13]. Moreover, several species of Selaginellaceae are commonly found together in a given habitat. All these factors make it difficult to identify these species by traditional morphological methods. Therefore, a method for the simple and accurate authentication of plants of the family Selaginellaceae is urgently needed to ensure the correct and safe use of these plants.

In taxonomy, the need for species identification at the genetic level has been increasingly recognized. The term “DNA barcode” was first coined by Hebert [14] in 2003. DNA barcoding is a process that uses a short DNA sequence from a standard locus as a species identification tool [15]. In 2009, a large consortium of researchers, *the Consortium for the Barcode of Life* (*CBOL*) *Plant Working Group*, proposed portions of two coding regions from the plastid (chloroplast) genome, *rbcL* and *matK*, as a “core barcode” for plant identification; these regions are supplemented with additional regions as required [16]. The *China Plant BOL Group* (*CPBG*) confirmed that the ITS/ITS2 regions should be incorporated into the core barcode for seed plants [17], [18]. Significant progress has been made in the identification of plants used in Chinese traditional medicine. Several DNA barcodes (*matK*, *rbcL*, *psbA-trnH*, ITS, ITS2, *rpoC1*, etc.) have been tested for the identification of species [19], [20], [21]. Furthermore, Chen *et al.* demonstrated the ability of ITS2 to discriminate more than 6,600 plant samples belonging to 4,800 species from 753 distinct genera and found that the rate of successful identification using the ITS2 barcode was 92.7% at the species level [20].

However, there has been no report of the use of the ITS2 barcode to identify medicinal plants of the Selaginellaceae family. Here, we validated the potential of the ITS2 region for the identification of closely related species of the Selaginellaceae family. Our study indicated that the ITS2 region could be used as an effective barcode for the identification of medicinal plants in Selaginellaceae.

## Materials and Methods

### Ethics Statement

All of our specimens were not collected from any national parks or protected areas, thus not requiring any specific permits for sampling. Specimens were collected from open areas, and they are not endangered or protected species.

### Plant Materials

A total of 103 samples belonging to 34 species of the Selaginellaceae family were collected from the main distribution areas in China; the geographical distributions and GenBank accession numbers are listed in Table S1. The origins of these samples covered 32 regions, including the Guizhou, Yunnan, Sichuan, Guangxi, Jiangsu, and Anhui provinces. Samples from almost all medicinal plants of the family Selaginellaceae, such as *S. tamariscina* and *S. puluinata*, which are included in the Chinese Pharmacopoeia [22], and *S. sinensis*, which is endemic to China [23], were collected. All plant species were identified by Professor Peishan Wang at the Institute of Biology, Guizhou Academy. The voucher samples were deposited in the herbarium of the Nanjing University of Chinese Medicine and the Guiyang College of Traditional Chinese Medicine.

### DNA Extraction, Amplification, and Sequencing

Genomic DNA was extracted from ∼10 mg silica gel-dried leaves according to the protocol provided with the Plant Genomic DNA Kit (Tiangen Biotech Co., China). The ITS2 region was amplified using the following pair of universal primers [24]: ITS-S2F (forward), 5′-ATGCGATACTTGGTGTGAAT, and ITS-S3R (reverse), 5′-GACGCTTCTCCAGACTACAAT. Primers were synthesized by Generay Co. (China). Polymerase chain reaction (PCR) amplification of the ITS2 region was performed using approximately 30 ng genomic DNA as a template in a 25 µL reaction mixture (2.5 µL 10× PCR buffer without MgCl_2_, 2 µL 25 mM MgCl_2_, 2 µL of each dNTP (2.5 mM), 1.0 µL of each primer (2.5 µM)), and 1.0 U of *Taq* DNA Polymerase. The reactions were performed with the following cycling conditions: 94°C for 5 min and 40 cycles of 94°C for 30 s, 56°C for 30 s, and 72°C for 45 s, followed by 72°C for 10 min. After the reactions, the samples were sequenced by Sangon Biotech (Shanghai).

### Data Analysis

Contig assembly and the generation of consensus sequences were performed using CodonCode Aligner V2.06 (CodonCode Co., USA). The ITS2 sequences were subjected to Hidden Markov Model (HMM) model [25] analysis to remove the conserved 5.8S and 28S DNA sequences [26]. The sequences of the DNA barcodes were aligned using Clustal W [27], and the genetic distances were computed using MEGA 5.1 [28] according to the Kimura 2-Parameter (K2P) model. Wilcoxon two-sample tests were performed to test the analysis results [27]. The distribution of intra- versus interspecific variability was compared using the DNA barcoding gaps based on K2P, and the presence or absence of the barcoding gap was determined from the sequence difference of *D* (*d*_inter_/*d*_intra_), computed by GraphPad Prism 5. Neighbor-joining (NJ) was conducted using MEGA 5.1 and was performed with 1,000 bootstrap replicates. Two methods of species identification, namely BLAST1 and the nearest distance method [29], were used to evaluate the species identification efficiency. Intraspecific sequence comparison was performed by searching the variable sites using MEGA 5.1. ITS2 sequences with different sequence divergence were subjected to secondary structure prediction in Selaginellaceae using tools from the ITS2 database [26].

## Results

### PCR Amplification Success Rate and Sequence Characteristics

**Figure 2:**
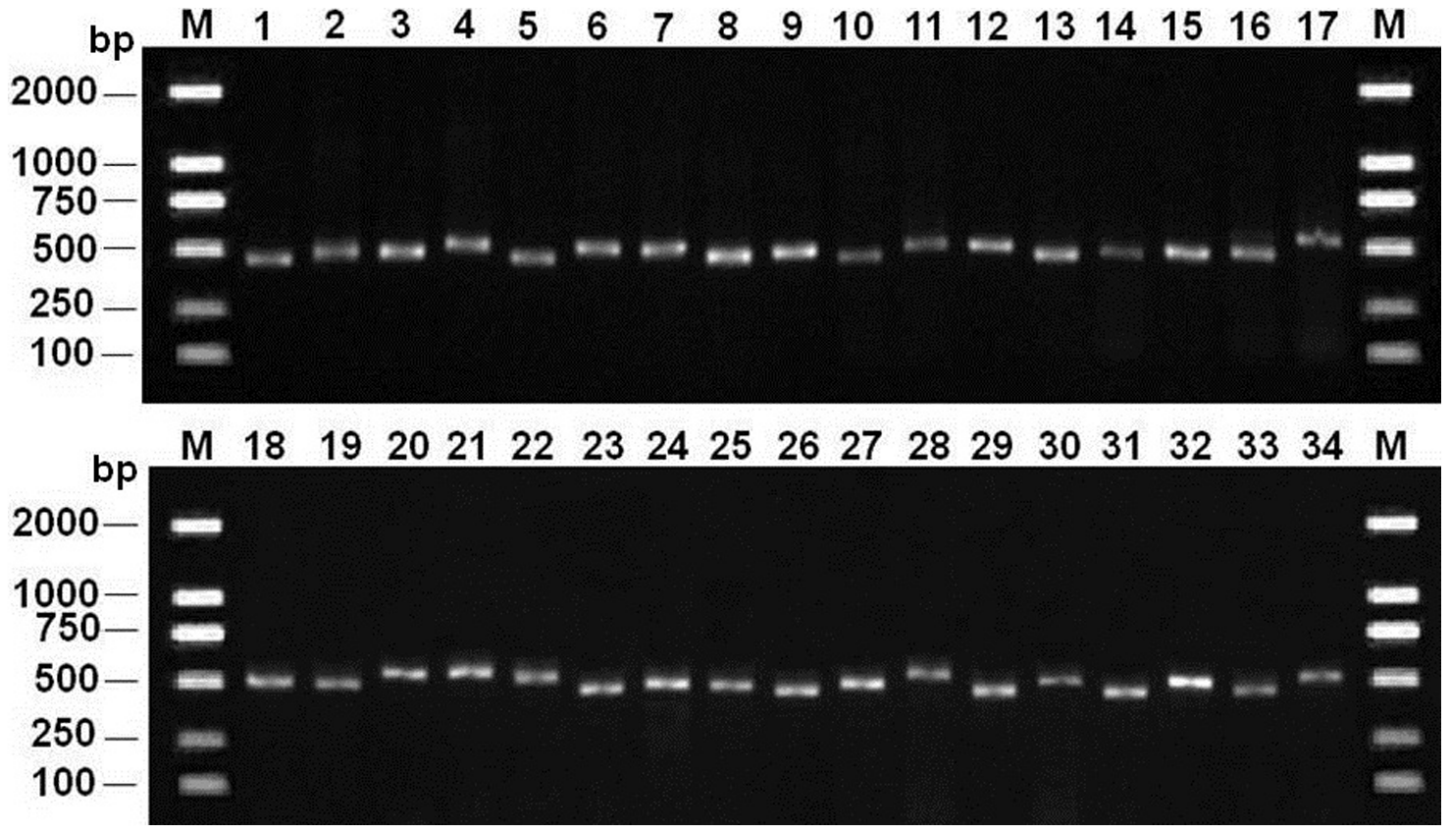
Electrophoretogram of PCR products from the ITS2 region of all the species in Selaginellaceae. 1- *S. amblyphylla* KC559782, 2-*S. bodinieri* KC559784, 3-*S. braunii* KC559786, 4-*S. chaetoloma* KC559787, 5-*S. chrysocaulos* KC559789, 6-*S. delicatula* KC559792, 7-*S. doederleinii* KC559798, 8-*S. drepanophylla* KC559800, 9-*S. effusa* KC559804, 10-*S. frondosa* KC559808, 11-*S. gebaueriana* KC559810, 12-*S. helferi* KC559812, 13-*S. heterostachys* KC559813, 14-*S. involvens* KC559819, 15-*S. kouycheensis* KC559827, 16-*S. kraussiana* KC559828, 17-*S. labordei* KC559830, 18-*S. leptophylla* KC559831, 19-*S. moellendorffii* KC559832, 20-*S. nipponica* KC559835, 21-*S. ornata* KC559841, 22-*S. picta* KC559843, 23-*S. pseudopaleifera* KC559844, 24-*S. pulvinata* KC559845, 25-*S. remotifolia* KC559851, 26-*S. repanda* KC559855, 27-*S. sanguinolenta* KC559857, 28-*S. siamensis* KC559859, 29-*S. sinensis* KC559861, 30-*S. tamariscina* KC559863, 31-*S. uncinata* KC559867, 32-*S. vardei* KC559879, 33-*S. willdenowii* KC559880, 34-*S. xipholepis* KC559883.

**Figure 3:**
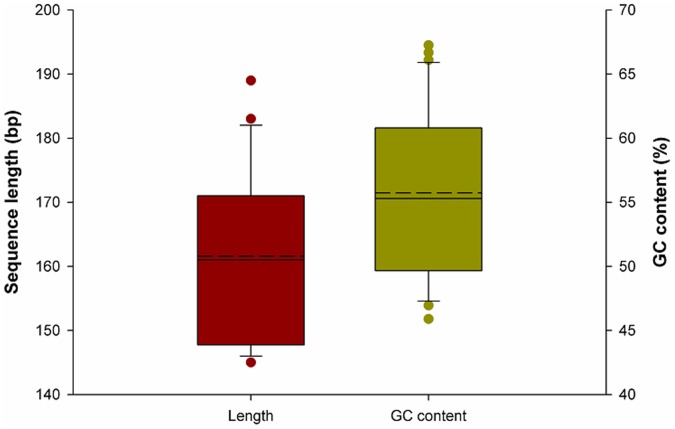
Distribution of sequence lengths and GC content of the ITS2 haplotypes from all Selaginellaceae species. The box represents the IQR of the data, which is defined as the difference between the 75th percentile and the 25th percentile. The solid lines in the middle of the box represent the median, and the dotted lines represent the average.

The PCR amplification rate of the ITS2 sequences from medicinal plants of Selaginellaceae was 100%, and the sequencing success rate was 100%. The amplified sequence length ranged from 450 to 550 bp (Figure 2). After removing the conserved 5.8S and 28S rRNA sequences, the lengths of the ITS2 sequences used in the analyses ranged from 145 to 189 bp, with an average length of 162 bp (Figure 3). The GenBank accession numbers are listed in Table S1. The mean GC content was 56% and ranged from 46% to 67% (Figure 3). Therefore, the length and GC content of Selaginellaceae ITS2 sequences are relatively variable.

### Genetic Divergence within and between Species

We estimated the genetic divergences of 103 samples using MEGA 5.1. Table 1 shows the calculated interspecific divergence, intraspecific divergence, smallest interspecific divergence, and largest intraspecific divergence. When calculated according to the K2P model, the intraspecific genetic distance (0.0289) is far less than the interspecific genetic distance (0.7259). The Wilcoxon two-sample tests results demonstrated that the differences between the interspecific and intraspecific divergences were significant (Table S2).

**Table 1:** Analyses of interspecific divergence and intraspecific variations of the ITS2 sequences in Selaginellaceae.

Measurement	K2P Value
All interspecific distance	0.7259±0.2041
The minimum interspecific distance	0.2507±0.2063
All intraspecific distance	0.0289±0.0713
Coalescent depth	0.0597±0.1200

### Assessment of Barcoding Gap

Based on the K2P model of intra- versus interspecific variation, the distribution frequencies in the medicinal plants of Selaginellaceae were analyzed on a scale of 0.002 distance units (Figure S1). The results indicated that the barcoding gap between interspecific and intraspecific divergence was obvious, and the proportion of interspecific genetic distance less than 0.03 was only 0.14%.

In addition, the sequence differences of *D* (*d*_inter_/*d*_intra_) were used to analyze the existence or absence of the barcoding gap, as shown in Figure S2. All values of *D* are clearly above the 1∶1 line, which is ideal because it indicates a discrete boundary between species of Selaginellaceae.

### The Efficacy of ITS2 for Authentication

The BLAST1 and nearest distance methods were used to evaluate the ability of the barcoding sequences in the given samples. The results showed ITS2 possessed 100% identification success rates at the species level for both methods. Overall, our study demonstrated that ITS2 was efficient and effective.

**Figure 4:**
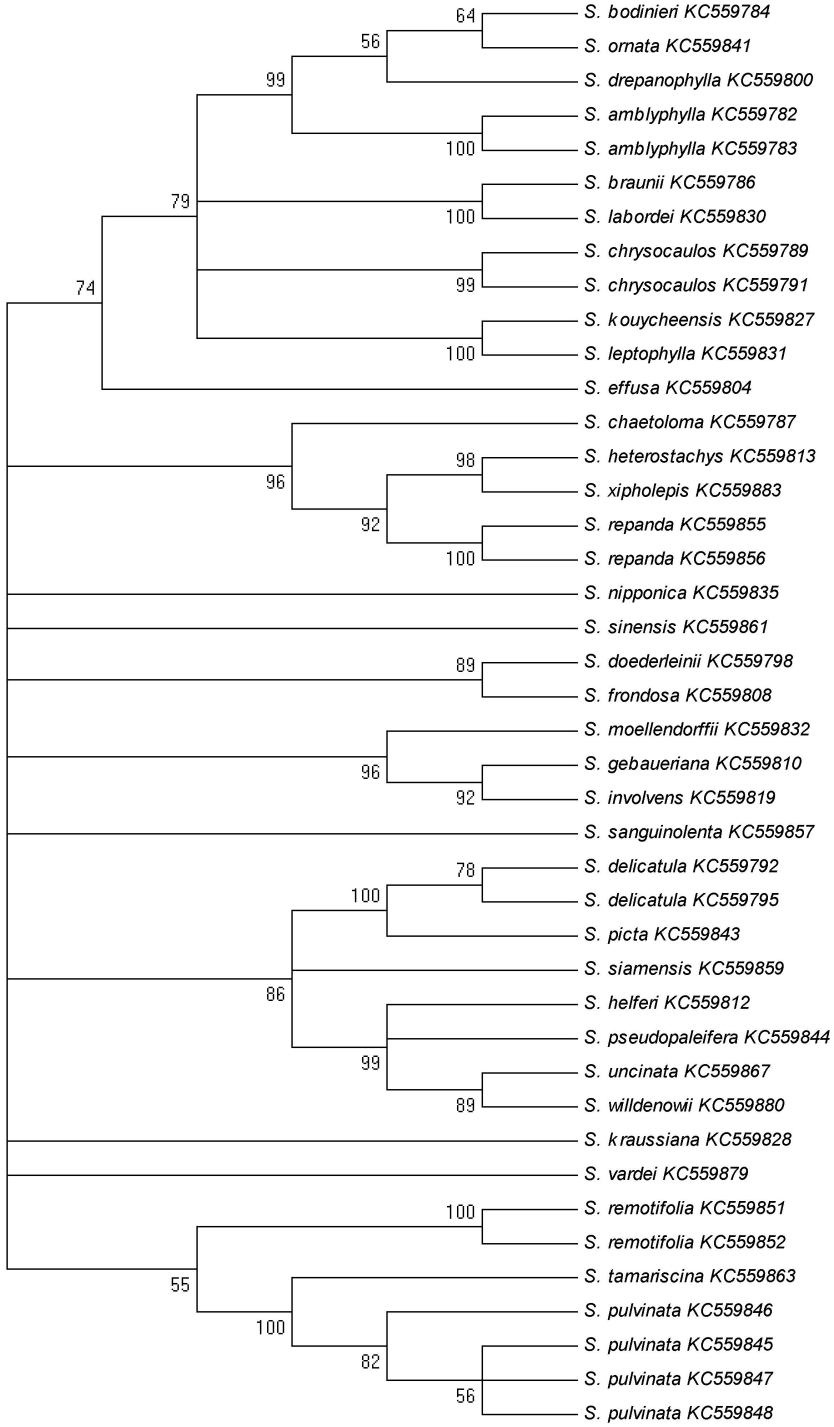
The NJ Tree constructed from the ITS2 haplotypes of Selaginellaceae. The percentage of replicate trees in which the associated taxa clustered together in 1000 bootstraps is shown next to the branches; values less than 50% were omitted.

The NJ tree intuitively displayed the relationship among the species in the Selaginellaceae family (Figure 4). More than one haplotype from the same species clustered into one branch, and there was a clear boundary between species. Interestingly, *S*. *tamariscina* and *S*. *pulvinata* are both listed in the Chinese pharmacopoeia and are more closely related than other species. The results were basically consistent with the traditional plant morphotaxonomy.

### Haplotypes and Secondary Structure of ITS2

Based on the alignments of the primary sequences of the ITS2 regions of the medicinal plants of Selaginellaceae, the statistics of the haplotypes and variable sites were calculated (Table S1 and Table 2). While six species had two or four haplotypes, 28 species had only one haplotype, although 20 of the 28 species had more than one sample. Among all samples, *S. amblyphylla*, *S. delicatula*, and *S. remotifolia* had two haplotypes with one variable site, *S. chrysocaulos* had two haplotypes with two variable sites, *S. repanda* had two haplotypes with three variable sites, and *S. pulvinata* had four haplotypes with four variable sites.

**Table 2:** The intraspecific variable sites in the ITS2 regions of Selaginellaceae.

Species	Haplotype	Sites (bp)	Base mutation
*S. amblyphylla*	A1	73	T
	A2	73	C
*S. chrysocaulos*	E1	16, 96	G, C
	E2	16, 96	A, T
*S. delicatula*	F1	19	A
	F2	19	T
*S. pulvinata*	X1	13, 116, 146, 170	T, G, A, C
	X2	13, 116, 146, 170	T, T, T, T
	X3	13, 116, 146, 170	T, G, T, C
	X4	13, 116, 146, 170	C, G, T, C
*S. remotifolia*	Y1	1	A
	Y2	1	T
*S. repanda*	Z1	262, 263, 269	A, A, A
	Z2	262, 263, 269	G, C, T

**Figure 5:**
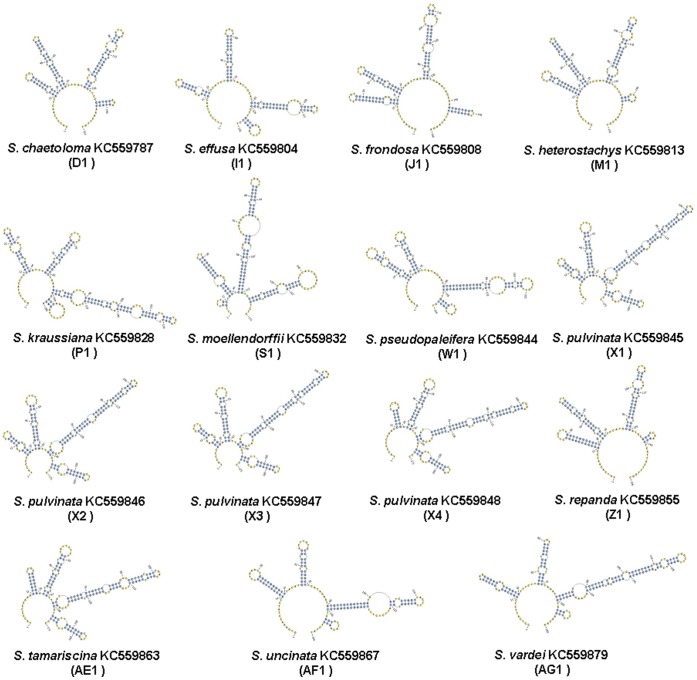
The secondary structures of the ITS2 regions in fifteen species of Selaginellaceae.

To identify the effect of the primary sequence divergences, secondary structures were constructed (Figure 5). All of the secondary structures of ITS2 in these species contained a central ring (primary ring) and four similar helices (I, II, III, and IV). However, ITS2 secondary structures among the different medicinal plants in Selaginellaceae differed significantly in the four helical regions in stem loop number, size, position, and screw angle. The secondary structures of the ITS2 sequences for some species could not be displayed because they had no reference models. On the basis of the ITS2 secondary structure, the medicinal plants of the Selaginellaceae family could be discriminated well.

## Discussion

Selaginellaceae family has a wide geographic distribution that includes an impressive range of habitats, including desert, tropical rainforest, alpine, and arctic habitats [30]. These species are important nonseed plants that possess a highly ecological and evolutionary significance. Several studies have used DNA barcoding methods to identify pteridophytes (ferns and lycophytes) [31], [32], [33]. Some researchers evaluated the potential to differentiate closely related sister species using DNA barcodes. They found that plastid barcoding regions could be useful to distinguish closely related taxa among the species whose relationships are still unclear and the corresponding boundaries among them are weak [34], [35], [36]. In previous studies, these plastid barcoding loci were thought to be suitable as DNA markers for species identification in medicinal pteridophytes. However, these reports tested plastid DNA barcode regions such as *rbcL*, *matK*, *trnH-psbA*, *trnL-F*, *rpoB*, and *rpoC1*, and no nuclear genes have been investigated in detail. In this study, we used the ITS2 region of nuclear ribosomal DNA to test 103 samples of 34 Selaginellaceae species. Our results highlighted the advantages of using the ITS2 region as a DNA barcode; these advantages include good universality, small intraspecific variation but high interspecific divergence, and a small fragment length, approximately 200 bp. Indeed, these advantages lead to easy amplification and sequencing in one Sanger reaction. In addition, not only did the primary sequences of ITS2 perform well in identifying Selaginellaceae species, but the secondary structures of ITS2 also provided sufficient molecular morphological characteristics to distinguish the Selaginellaceae species. Recently, an increasing number of studies suggest that DNA secondary structures are crucial for genomic stability and cellular processes, such as transcription [37], [38]. Therefore, the benefits of the use of the ITS2 to identify Selaginellaceae species may be twofold. plants of the family Selaginellaceae contain unique secondary metabolic pathways, and different species have different secondary metabolic products, leading to discrepancies in their clinical application [39]. Because Selaginellaceae species are often traded internationally for their medical and ornamental importance, our research provides a convenient tool to validate Selaginellaceae species. Previously, the ITS2 region was confirmed as a novel barcode for identifying medicinal plant species [20], [24], [27], [40], but this study expanded the application of the ITS2 region to the medicinal plant field. The *China Plant BOL Group* has proposed that ITS/ITS2 should be incorporated into the core barcode for seed plants [17], and our research has broadened the application of the ITS2 region to nonseed plants.

Plants of Selaginellaceae are traditionally identified based on their morphological characteristics, including spores and leaf margin [13], [41]. However, morphological identification by taxonomic experts depends on sufficient experience and can easily be affected by geographical environment and biocoenosis [42], [43]. As a result, ecological and phylogenetic studies of Selaginellaceae have been limited. DNA barcode technology is widely used because genomic sequence is not influenced by individual characteristics and developmental stages and the procedure is relatively simple [44]. DNA barcoding is an effective supplement to traditional morphological methods. Molecular phylogenetic research in the Selaginellaceae family has been limited. The plastid *rbcL* gene and the nuclear 26S ribosomal DNA gene have extremely high substitution rates [45], [46]. In our study, similar results were obtained, i.e., the genetic divergence of ITS2 between the species of Selaginellaceae was high. However, we found that the genetic divergence of ITS2 within Selaginellaceae species was small; most species had two or more samples. Actually, most species had only one haplotype with no intraspecific variation. Previous studies used only one sample for each species and could not determine the intraspecific divergence. According to our neighbor-joining tree created from the ITS2 sequences in Selaginellaceae, different haplotypes from the same species converged into one branch. Hence, analysis of the ITS2 region may contribute to the phylogenetic analysis of Selaginellaceae. In addition, *S. tamariscina* and *S. pulvinata* are closely related according to the neighbor-joining tree, suggesting that their genetic relationship is relatively intimate. This evidence supports the inclusion of these two species in the Chinese pharmacopoeia as the origins of the Herba Selaginellae.

In 2009, Smith reported a higher GC content in the mitochondrial DNA (mtDNA) and plastid DNA (ptDNA) of Selaginellaceae than that in organellar DNA in other plant taxa, and both of these nucleotide biases are influenced by the high levels of RNA editing that occur in the organelles [30]. However, the GC content of the *S. moellendorffii* nuclear genome is ∼63%, which is similar to other land plants [30]. Our results indicate that in Selaginellaceae, the mean content of the ITS2 barcode, a small nuclear rDNA fragment, is 56%, which is close to the content of ferns but lower than that of other land plants [24]. One notable aspect is that the nuclear DNA of land plants, unlike its organellar DNA, is believed to undergo very little RNA editing. Thus, understanding the discordance between the nucleotide composition of the nrDNA ITS2 region and organelle DNA may have wide-reaching evolutionary implications.

### Conclusions

We confirmed that the ITS2 region can be used as a universal barcode to distinguish the medicinal plants of the Selaginellaceae family, a nonseed plant taxon with ecological, evolutionary, and medical importance. The successful rate of PCR amplification and sequencing of the ITS2 region was 100%. The lengths of the ITS2 regions ranged from 145 to 189 bp, with an average length of 162 bp; the mean GC content was 56%, with a range of 46% to 67%. There was significant divergence between the interspecific and intraspecific genetic distances of the ITS2 regions, while the barcoding gap was more obvious. The helical regions of the ITS2 secondary structures in Selaginellaceae were significantly different in stem loop number, size, position and screw angle. Cluster analysis using the ITS2 barcode in Selaginellaceae was basically consistent with traditional plant morphology. This study broadened the application of the ITS2 region in the medicinal field and in nonseed plants and provided solid evidence that the ITS2 region has the potential to contribute to the phylogenetic analysis of Selaginellaceae.

## Supporting Information

Figure S1 **Distribution of the intra- and interspecific variations of the ITS2 regions in Selaginellaceae.**
(TIF)

Figure S2 **Existence or absence of the barcoding gap based on the K2P distance in Selaginellaceae.**
(TIF)

Table S1 **Plant samples of Selaginellaceae used in the present study.**
(DOC)

Table S2 **Wilcoxon two-sample tests for the distribution of intra- vs. interspecific divergences.**
(DOC)
