# Supplementary figures and images for: Application of the ITS2 Region for Barcoding Medicinal Plants of Selaginellaceae in Pteridophyta
Source: PLoS One. 2013 Jun 27;8(6):e67818. doi: 10.1371/journal.pone.0067818 (PMC3694882; doi:10.1371/journal.pone.0067818)

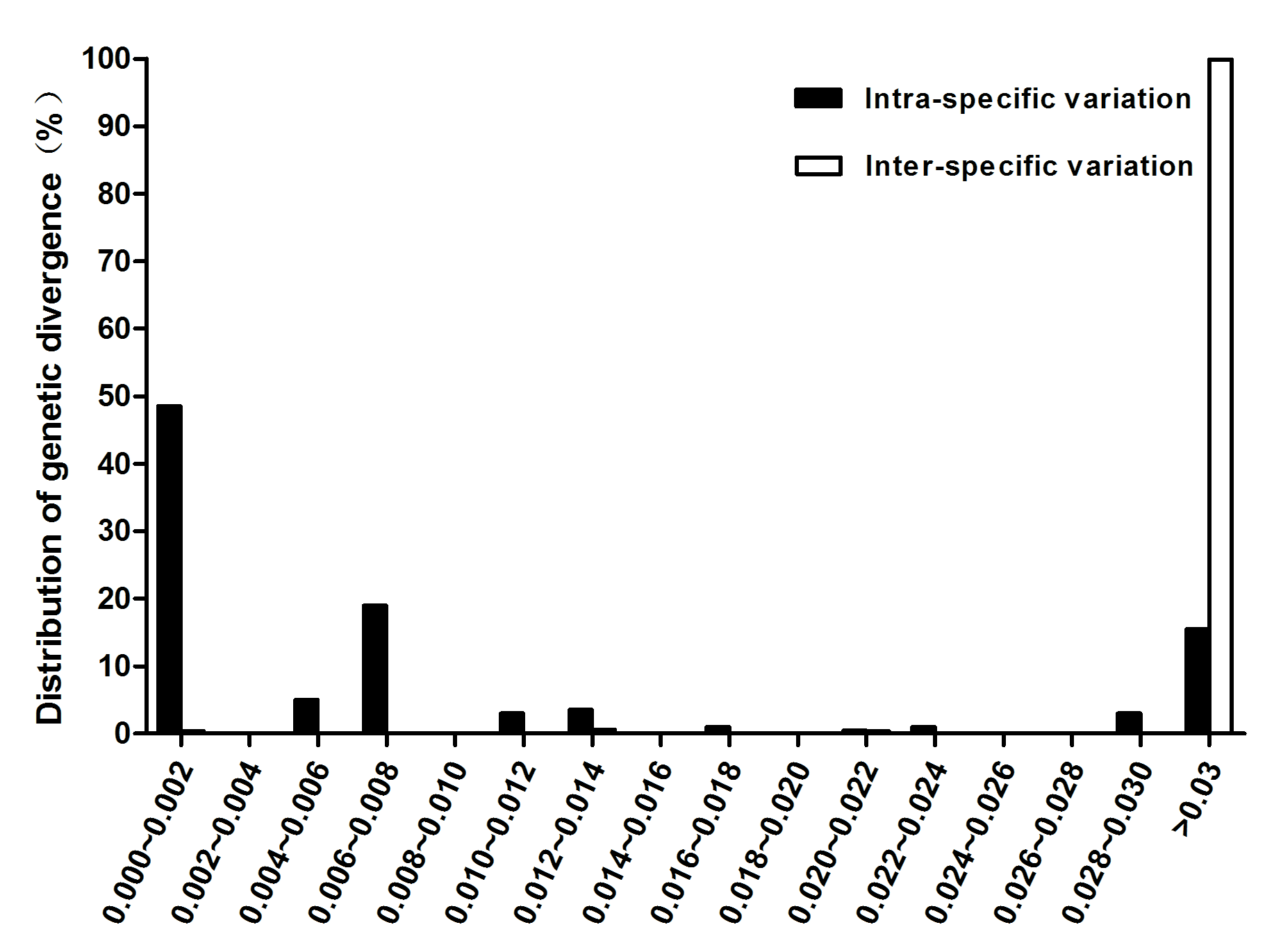

Supplement: Figure S1 — Distribution of the intra- and interspecific variations of the ITS2 regions in Selaginellaceae. (TIF) [file pone.0067818.s001.tif]

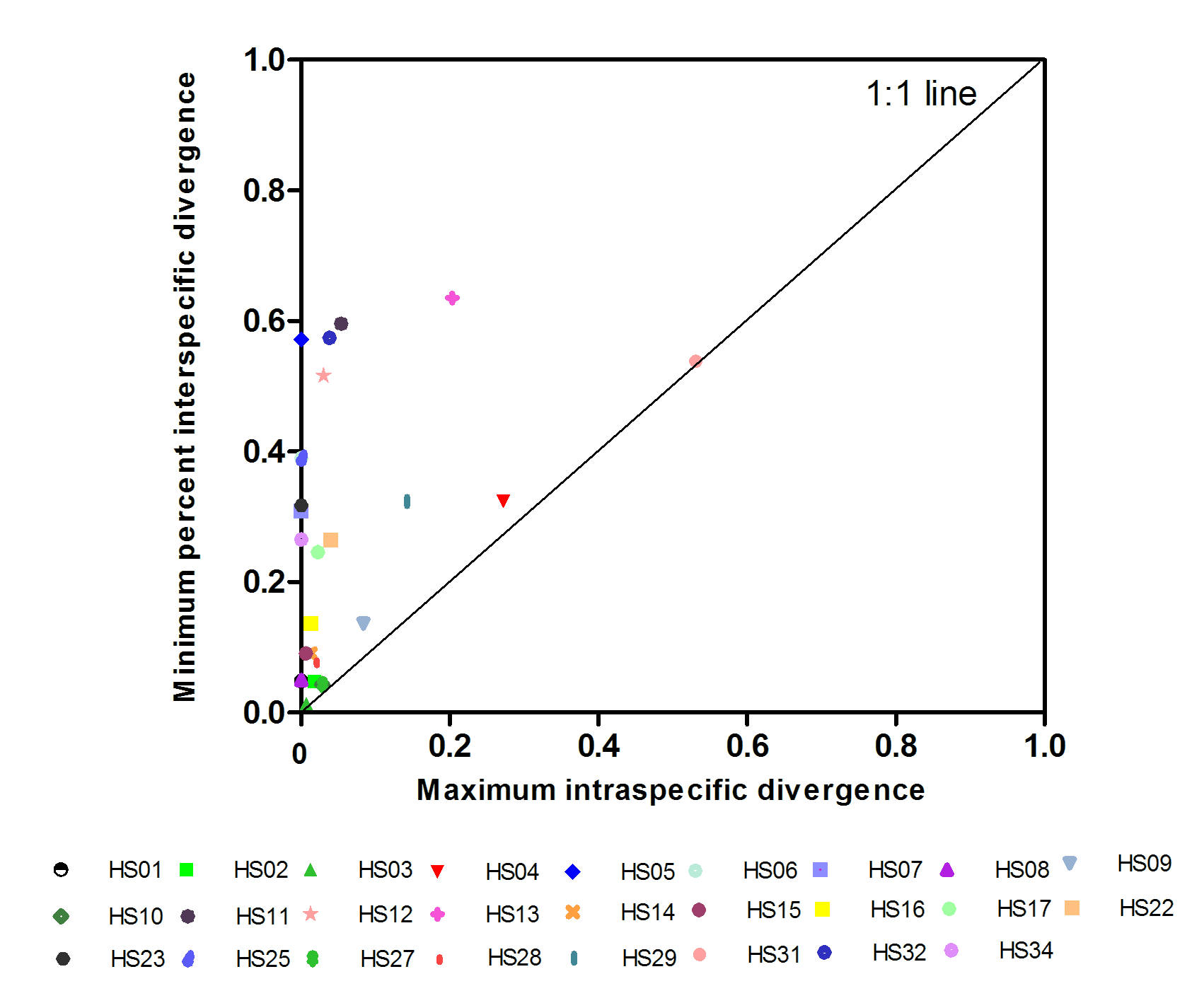

Supplement: Figure S2 — Existence or absence of the barcoding gap based on the K2P distance in Selaginellaceae. (TIF) [file pone.0067818.s002.tif]
