# Supplementary material for: Application of the ITS2 Region for Barcoding Medicinal Plants of Selaginellaceae in Pteridophyta
Source: PLoS One. 2013 Jun 27;8(6):e67818. doi: 10.1371/journal.pone.0067818 (PMC3694882; doi:10.1371/journal.pone.0067818)
Supplement: Table S1 — Plant samples of Selaginellaceae used in the present study. (DOC) [file pone.0067818.s003.doc]

**Table S1.** Plant samples of Selaginellaceae used in the present study.

| Species | Haplotype | Collection site | Voucher number | GenBank Accession No. |
| --- | --- | --- | --- | --- |
| *Selaginella amblyphylla* | A1 | Puwen, Yunnan | HS0101 | KC559782 |
| *S. amblyphylla* | A2 | Puwen, Yunnan | HS0102 | KC559783 |
| *S. bodinieri* | B1 | Libo, Guizhou | HS0201 | KC559784 |
| *S. bodinieri* | B1 | Libo, Guizhou | HS0202 | KC559785 |
| *S. braunii* | C1 | Songtao, Guizhou | HS0301 | KC559786 |
| *S. chaetoloma* | D1 | Luocheng, Guangxi | HS0401 | KC559787 |
| *S. chaetoloma* | D1 | Luocheng, Guangxi | HS0402 | KC559788 |
| *S. chrysocaulos* | E1 | Hezhang, Guizhou | HS0501 | KC559789 |
| *S. chrysocaulos* | E1 | Hezhang, Guizhou | HS0502 | KC559790 |
| *S. chrysocaulos* | E2 | Dali, Yunnan | HS0503 | KC559791 |
| *S. delicatula* | F1 | Liuchongguan, Guizhou | HS0601 | KC559792 |
| *S. delicatula* | F1 | Liuchongguan, Guizhou | HS0602 | KC559793 |
| *S. delicatula* | F1 | Libo, Guizhou | HS0603 | KC559794 |
| *S. delicatula* | F2 | Libo, Guizhou | HS0604 | KC559795 |
| *S. delicatula* | F1 | Libo, Guizhou | HS0605 | KC559796 |
| *S. delicatula* | F2 | Libo, Guizhou | HS0606 | KC559797 |
| *S. doederleinii* | G1 | Wujiahe, Guizhou | HS0701 | KC559798 |
| *S. doederleinii* | G1 | Libo, Guizhou | HS0702 | KC559799 |
| *S. drepanophylla* | H1 | Libo, Guizhou | HS0801 | KC559800 |
| *S. drepanophylla* | H1 | Libo, Guizhou | HS0802 | KC559801 |
| *S. drepanophylla* | H1 | Libo, Guizhou | HS0803 | KC559802 |
| *S. drepanophylla* | H1 | Libo, Guizhou | HS0804 | KC559803 |
| *S. effusa* | I1 | Wujiahe, Guizhou | HS0901 | KC559804 |
| *S. effusa* | I1 | Wujiahe, Guizhou | HS0902 | KC559805 |
| *S. effusa* | I1 | Wujiahe, Guizhou | HS0903 | KC559806 |
| *S. effusa* | I1 | Wujiahe, Guizhou | HS0904 | KC559807 |
| *S. frondosa* | J1 | Menglamenglun, Yunnan | HS1001 | KC559808 |
| *S. frondosa* | J1 | Menglamenglun, Yunnan | HS1002 | KC559809 |
| *S. gebaueriana* | K1 | Puer, Yunnan | HS1101 | KC559810 |
| *S. gebaueriana* | K1 | Puer, Yunnan | HS1102 | KC559811 |
| *S. helferi* | L1 | Mengyang, Yunnan | HS1201 | KC559812 |
| *S. heterostachys* | M1 | Guiyang, Guizhou | HS1301 | KC559813 |
| *S. heterostachys* | M1 | Guiyang, Guizhou | HS1302 | KC559814 |
| *S. heterostachys* | M1 | Ebian, Sichuan | HS1303 | KC559815 |
| *S. heterostachys* | M1 | Ebian, Sichuan | HS1304 | KC559816 |
| *S. heterostachys* | M1 | Libo, Guizhou | HS1305 | KC559817 |
| *S. heterostachys* | M1 | Libo, Guizhou | HS1306 | KC559818 |
| *S. involvens* | N1 | Libo, Guizhou | HS1401 | KC559819 |
| *S. involvens* | N1 | Libo, Guizhou | HS1402 | KC559820 |
| *S. involvens* | N1 | Libo, Guizhou | HS1403 | KC559821 |
| *S. involvens* | N1 | Libo, Guizhou | HS1404 | KC559822 |
| *S. involvens* | N1 | Libo, Guizhou | HS1405 | KC559823 |
| *S. involvens* | N1 | Libo, Guizhou | HS1406 | KC559824 |
| *S. involvens* | N1 | Yixing, Jiangsu | HS1407 | KC559825 |
| *S. involvens* | N1 | Yixing, Jiangsu | HS1408 | KC559826 |
| *S. kouycheensis* | O1 | Libo, Guizhou | HS1501 | KC559827 |
| *S.* *kraussiana* | P1 | Guiyang, Guizhou | HS1601 | KC559828 |
| *S.* *kraussiana* | P1 | Guiyang, Guizhou | HS1602 | KC559829 |
| *S. labordei* | Q1 | Anlong, Guizhou | HS1701 | KC559830 |
| *S. leptophylla* | R1 | Ziyun, Guizhou | HS1801 | KC559831 |
| *S. moellendorffii* | S1 | Yashan, Anhui | HS1901 | KC559832 |
| *S. moellendorffii* | S1 | Tianmushan, Zhejiang | HS1902 | KC559833 |
| *S. moellendorffii* | S1 | Tianmushan, Zhejiang | HS1903 | KC559834 |
| *S. nipponica* | T1 | Libo, Guizhou | HS2001 | KC559835 |
| *S. nipponica* | T1 | Libo, Guizhou | HS2002 | KC559836 |
| *S. nipponica* | T1 | Libo, Guizhou | HS2003 | KC559837 |
| *S. nipponica* | T1 | Yashan, Anhui | HS2004 | KC559838 |
| *S. nipponica* | T1 | Tianmushan, Zhejiang | HS2005 | KC559839 |
| *S. nipponica* | T1 | Tianmushan, Zhejiang | HS2006 | KC559840 |
| *S. ornata* | U1 | Puwen, Yunnan | HS2101 | KC559841 |
| *S. ornata* | U1 | Jinghong, Yunnan | HS2102 | KC559842 |
| *S. picta* | V1 | Menglamenglun, Yunnan | HS2201 | KC559843 |
| *S. pseudopaleifera* | W1 | Lingping, Yunnan | HS2301 | KC559844 |
| *S. pulvinata* | X1 | Puer, Yunnan | HS2401 | KC559845 |
| *S. pulvinata* | X2 | Puer, Yunnan | HS2402 | KC559846 |
| *S. pulvinata* | X3 | Weining, Guizhou | HS2403 | KC559847 |
| *S. pulvinata* | X4 | Weining, Guizhou | HS2404 | KC559848 |
| *S. pulvinata* | X3 | Longan, Guangxi | HS2405 | KC559849 |
| *S. pulvinata* | X3 | Longan, Guangxi | HS2406 | KC559850 |
| *S. remotifolia* | Y1 | Libo, Guizhou | HS2501 | KC559851 |
| *S. remotifolia* | Y2 | Libo, Guizhou | HS2502 | KC559852 |
| *S. remotifolia* | Y1 | Liuchongguan, Guizhou | HS2503 | KC559853 |
| *S. remotifolia* | Y2 | Liuchongguan, Guizhou | HS2504 | KC559854 |
| *S. repanda* | Z1 | Mengyuan, Yunnan | HS2601 | KC559855 |
| *S. repanda* | Z2 | Longling, Yunnan | HS2602 | KC559856 |
| *S. sanguinolenta* | AB1 | Panxian, Guizhou | HS2701 | KC559857 |
| *S. sanguinolenta* | AB1 | Panxian, Guizhou | HS2702 | KC559858 |
| *S. siamensis* | AC1 | Menglamenglun, Yunnan | HS2801 | KC559859 |
| *S. siamensis* | AC1 | Menglamenglun, Yunnan | HS2802 | KC559860 |
| *S. sinensis* | AD1 | Lianyungang, Jiangsu | HS2901 | KC559861 |
| *S. sinensis* | AD1 | Lianyungang, Jiangsu | HS2902 | KC559862 |
| *S. tamariscina* | AE1 | Lianyungang, Jiangsu | HS3001 | KC559863 |
| *S. tamariscina* | AE1 | Lianyungang, Jiangsu | HS3002 | KC559864 |
| *S. tamariscina* | AE1 | Luan, Anhui | HS3003 | KC559865 |
| *S. tamariscina* | AE1 | Luan, Anhui | HS3004 | KC559866 |
| *S. uncinata* | AF1 | Liuchongguan, Guizhou | HS3101 | KC559867 |
| *S. uncinata* | AF1 | Liuchongguan, Guizhou | HS3102 | KC559868 |
| *S. uncinata* | AF1 | Xishuangbanna, Kunming | HS3103 | KC559869 |
| *S. uncinata* | AF1 | Lihua, Guizhou | HS3104 | KC559870 |
| *S. uncinata* | AF1 | Lihua, Guizhou | HS3105 | KC559871 |
| *S. uncinata* | AF1 | Libo, Guizhou | HS3106 | KC559872 |
| *S. uncinata* | AF1 | Libo, Guizhou | HS3107 | KC559873 |
| *S. uncinata* | AF1 | Libo, Guizhou | HS3108 | KC559874 |
| *S. uncinata* | AF1 | Tianmushan, Zhejiang | HS3109 | KC559875 |
| *S. uncinata* | AF1 | Tianmushan, Zhejiang | HS3110 | KC559876 |
| *S. uncinata* | AF1 | Longan, Guangxi | HS3111 | KC559877 |
| *S. uncinata* | AF1 | Longan, Guangxi | HS3112 | KC559878 |
| *S. vardei* | AG1 | Ziyuan, Guizhou | HS3201 | KC559879 |
| *S. willdenowii* | AH1 | Wangmo, Guizhou | HS3301 | KC559880 |
| *S. willdenowii* | AH1 | Wangmo, Guizhou | HS3302 | KC559881 |
| *S. willdenowii* | AH1 | Wangmo, Guizhou | HS3303 | KC559882 |
| *S. xipholepis* | AI1 | Libo, Guizhou | HS3401 | KC559883 |
| *S. xipholepis* | AI1 | Libo, Guizhou | HS3402 | KC559884 |
