# Supplementary material for: Application of the ITS2 Region for Barcoding Medicinal Plants of Selaginellaceae in Pteridophyta
Source: PLoS One. 2013 Jun 27;8(6):e67818. doi: 10.1371/journal.pone.0067818 (PMC3694882; doi:10.1371/journal.pone.0067818)
Supplement: Table S2 — Wilcoxon two-sample tests for the distribution of intra- vs. interspecific divergences. (DOC) [file pone.0067818.s004.doc]

**Table S2.** Wilcoxon two-sample tests for the distribution of intra-vs. interspecific divergences.

| No. of interspecific distances | No. of intraspecific distances | Wilcoxon W | P value |
| --- | --- | --- | --- |
| 5053 | 200 | 23737.5 | 1.035×10^-125^ |
